# Species richness, distribution and genetic diversity of *Caenorhabditis* nematodes in a remote tropical rainforest

**DOI:** 10.1186/1471-2148-13-10

**Published:** 2013-01-12

**Authors:** Marie-Anne Félix, Richard Jovelin, Céline Ferrari, Shery Han, Young Ran Cho, Erik C Andersen, Asher D Cutter, Christian Braendle

**Affiliations:** 1 Institut de Biologie de l’Ecole Normale Supérieure, CNRS - ENS - INSERM, 46 rue d’Ulm, Paris cedex 05, 75230, France; 2 Department of Ecology and Evolutionary Biology, University of Toronto, 25 Willcocks St, Toronto, ON, M5S 3B2, Canada; 3 Institut de Biologie Valrose, CNRS, UMR7277, Parc Valrose, Nice cedex 02, 06108, France; 4 INSERM, U1091, Nice cedex 02, 06108, France; 5 University of Nice Sophia Antipolis, UFR Sciences, Nice cedex 02, 06108, France; 6 Department of Ecology and Evolutionary Biology, Lewis-Sigler Institute for Integrative Genomics, Princeton University, Princeton, NJ, USA

**Keywords:** *Caenorhabditi*s, Species richness, Population structure, *C. briggsae*, Nucleotide diversity

## Abstract

**Background:**

In stark contrast to the wealth of detail about *C. elegans* developmental biology and molecular genetics, biologists lack basic data for understanding the abundance and distribution of *Caenorhabditis* species in natural areas that are unperturbed by human influence.

**Methods:**

Here we report the analysis of dense sampling from a small, remote site in the Amazonian rain forest of the Nouragues Natural Reserve in French Guiana.

**Results:**

Sampling of rotting fruits and flowers revealed proliferating populations of *Caenorhabditis*, with up to three different species co-occurring within a single substrate sample, indicating remarkable overlap of local microhabitats. We isolated six species, representing the highest local species richness for *Caenorhabditis* encountered to date, including both tropically cosmopolitan and geographically restricted species not previously isolated elsewhere. We also documented the structure of within-species molecular diversity at multiple spatial scales, focusing on 57 *C. briggsae* isolates from French Guiana. Two distinct genetic subgroups co-occur even within a single fruit. However, the structure of *C. briggsae* population genetic diversity in French Guiana does not result from strong local patterning but instead presents a microcosm of global patterns of differentiation. We further integrate our observations with new data from nearly 50 additional recently collected *C. briggsae* isolates from both tropical and temperate regions of the world to re-evaluate local and global patterns of intraspecific diversity, providing the most comprehensive analysis to date for *C. briggsae* population structure across multiple spatial scales.

**Conclusions:**

The abundance and species richness of *Caenorhabditis* nematodes is high in a Neotropical rainforest habitat that is subject to minimal human interference. Microhabitat preferences overlap for different local species, although global distributions include both cosmopolitan and geographically restricted groups. Local samples for the cosmopolitan *C. briggsae* mirror its pan-tropical patterns of intraspecific polymorphism. It remains an important challenge to decipher what drives *Caenorhabditis* distributions and diversity within and between species.

## Background

Understanding the patterns and drivers that govern the abundance and diversity of organisms motivates the study of ecology and evolution. Historically, however, such study has focused primarily on non-model organisms, with some notable exceptions (e.g. *Drosophila melanogaster* in evolutionary biology). Ecological and evolutionary investigations have particularly neglected the nematode *Caenorhabditis elegans* and its close relatives until recently [1,2]. And yet, the deep mechanistic knowledge about *C. elegans* development and life history, coupled with its experimental advantages in the laboratory, make it particularly suited for a more complete integration of molecular genetic approaches with evolutionary and ecological questions. This merging of disciplines is motivated further by interesting phylogenetically variable characteristics of the genus, such as the independent origins of androdioecy (i.e. self-fertilizing reproduction by hermaphrodites and facultative outcrossing with males) in at least three lineages from the more prevalent gonochoristic reproductive mode among other *Caenorhabditis* species, which reproduce through obligatory mating of females and males [3,4]. A major impediment to fully realizing the potential of integrating these fields, however, is the lack of basic information about species distribution and richness in nature.

Studies of *Caenorhabditis* in nature are difficult, not only because of their small size (ca. 1 mm length), but also because we know little about the habitats in which they can be found reliably [2]. For this reason, the first population genetic analyses on *C. elegans* were conducted from opportunistic collection or by sampling populations in artificial compost heaps [5-10]. Subsequently, *C. elegans* was isolated from rotting fruits, as well as in phoretic association with invertebrates such as snails, slugs and isopods [3,5,11,12]. Compost heap populations are mostly composed of animals in dauer diapause, but *C. elegans* and *C. briggsae* were recently shown to proliferate and feed in rotting fruits and stems in mainland France [13]. Those rotting fruits were collected primarily in human-built orchards; although rotting stems are found in natural preserves, primary forests no longer exist in Europe [13]. Like many other model organisms (*Drosophila melanogaster*, *Saccharomyces cerevisiae*, *Mus musculus*), *C. elegans* is thus at least partly human-associated, which likely exerts a strong influence on its range, migration patterns, and population structure [5,14,15]. However, we still have very limited information on the distribution of *C. elegans* and other *Caenorhabditis* species relating to habitat type and geographic location. Most importantly, we lack information on *Caenorhabditis* species diversity and population structure from unperturbed habitats.

Here, we report the results of a systematic sampling of *Caenorhabditis* across spatial scales, from within a single fruit to sampling at metre, kilometre, regional and global scales. In order to minimize the impact of humans and to contrast previously studied temperate sites with a tropical location, we sampled *Caenorhabditis* populations in a primary Neotropical rain forest in French Guiana, at the Nouragues National Reserve. We quantified the structure of diversity at different genetic and spatial scales, including the species richness of *Caenorhabditis* at the Nouragues location. In addition, we examined levels of intraspecific molecular polymorphism of *C. briggsae* from French Guiana and from additional recent local sampling of *C. briggsae* from different regions of the world.

## Results

### Species diversity in French Guiana

**Figure 1:**
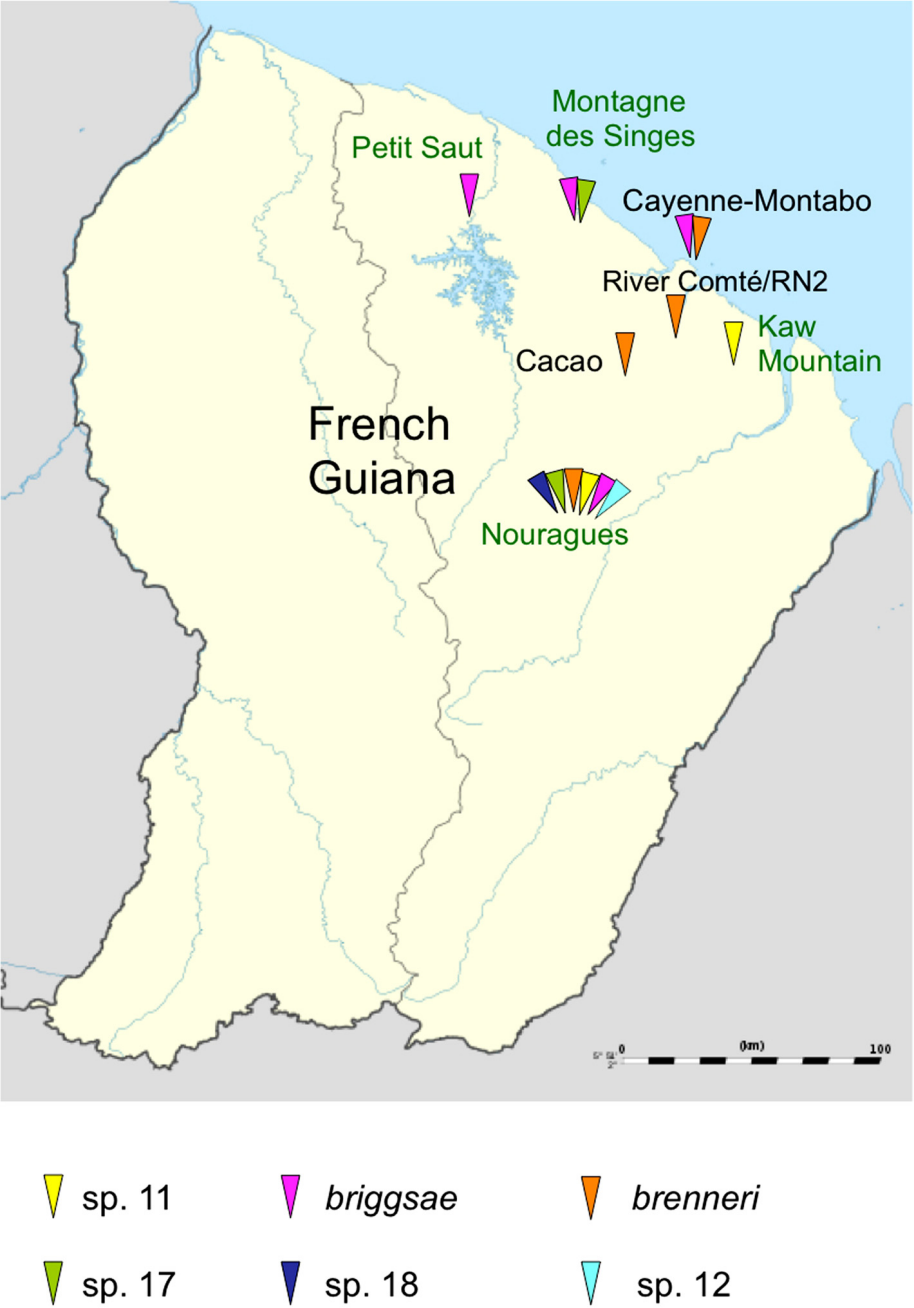
**Sampling of *Caenorhabditis *species in French Guiana. **The triangles indicate the presence of different *Caenorhabditis* species in the sampled areas, colour-coded as indicated in the legend. The names of locations with strong human activity (e.g. towns and villages) are written in black, and other locations (e.g. forests) in green.

**Figure 2:**
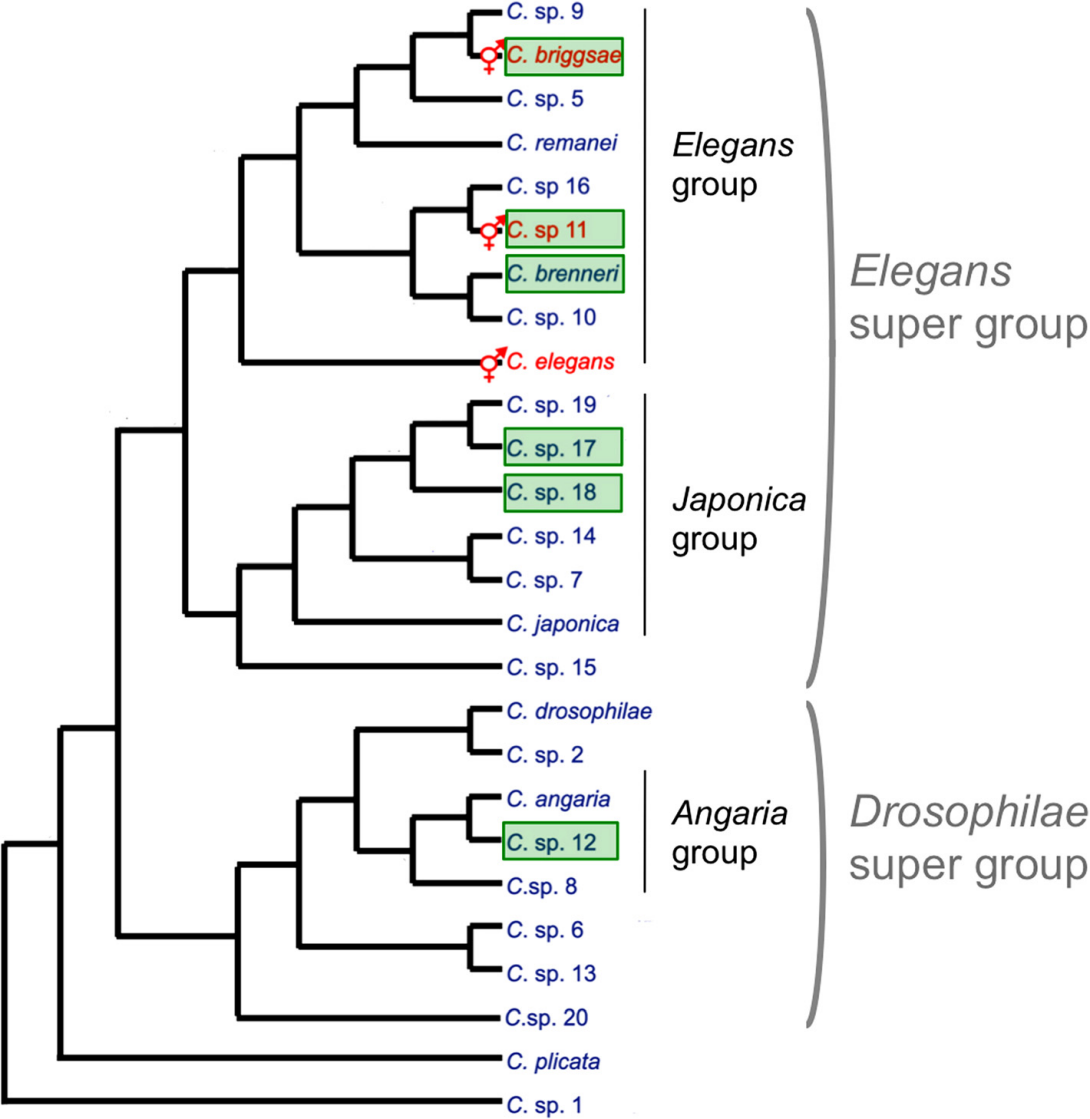
**Phylogeny of the *Caenorhabditis *genus, adapted from Kiontke *et al. *2011 **[3]**. **A green box highlights the species found in French Guiana. The selfing species are indicated in red.

We assessed a total of 184 samples for nematodes of the genus *Caenorhabditis* from a broad spectrum of substrate types in French Guiana (Figures 1 and 2, Table 1, Additional files 1 and 2). We isolated five different *Caenorhabditis* species, in addition to a sixth species (*C.* sp. 12) found in May 2008 that was not found in 2009. Three of these species (*C.* sp. 12, *C*. sp. 17, and *C.* sp. 18) have so far only been reported from French Guiana [3]; all three are gonochoristic. *C.* sp. 12 belongs to the *Drosophilae* supergroup of *Caenorhabditis* species, and is presently the sister species of *C. angaria*, with which it can form F1 hybrids [3]. *C.* sp. 17 and *C.* sp. 18 are closely related to each other within the *Japonica* group of *Caenorhabditis* (Figure 2). The other three species found in Nouragues are species known from tropical regions on other continents [3], namely, the gonochoristic *C. brenneri* and the two selfing androdioecious species, *C. briggsae* and *C.* sp. 11. The most commonly encountered species at the Nouragues Natural Reserve were the androdieocious species (*C. briggsae*, *C.* sp. 11) and the new gonochoristic species *C*. sp. 17.

**Table 1:** **Summary of *Caenorhabditis *species and isolates isolated from different localities in French Guiana**

**Species**	Mode of reproduction*	**Number of isolates**	**Locations in French Guiana (No. of isolates)**	**Known distribution**
*C. briggsae*	androdioecy	62	Nouragues (54), Montagne des Singes (6), Montabo (1), Petit Saut (1)	Cosmopolitan
*C*. sp. 11	androdioecy	31	Nouragues (30), Kaw mountain (1)	Cosmopolitan, tropical
*C. brenneri*	gonochorism	13	Nouragues (9), Montabo (1), Comté riverbank (1), Cacao (2)	Cosmopolitan, tropical
*C*. sp. 17	gonochorism	30	Nouragues (28), Montagne des Singes (2)	French Guiana
*C*. sp. 18	gonochorism	13	Nouragues (13)	Nouragues
*C*. sp. 12	gonochorism	2	Nouragues (2)	Nouragues

*androdioecy = self-fertile hermaphrodites and males, gonochorism = females and males.

### Substrate type and presence of *Caenorhabditis*

We found *Caenorhabditis* nematodes in a wide variety of rotting vegetal substrates (fruits, flowers, plant stems); however, we could not detect any obvious substrate specificity of the different species (Additional file 2). Samples examined immediately after isolation indicated that *Caenorhabditis* population size in a given sample varied greatly, from one to approximately one thousand individuals, including both dauer stages and proliferating populations with non-dauer juvenile and adult stages (Additional file 2). Of the 130 Nouragues samples, 33 (25%) contained *Caenorhabditis*, including 16 of 48 rotting fruits and 11 of 19 rotting flowers. Proliferating populations of *Caenorhabditis* were found both in rotting fruits and flowers, as well as in one of three samples of erect live flowers (*Heliconia* sp.). *Caenorhabditis* nematodes were rarely found in humus or leaf litter (3 of 24) or in association with live or dead insects (0 of ~20). In 8 of 33 positive samples, we found two or three different *Caenorhabditis* species co-occurring in rotting fruits or flowers sampled at the same site (e.g. *C. briggsae,* spp. 11 and 17, isolated from *Cecropia* fruits, sample D25, Additional file 2). Many samples also contained nematode species other than *Caenorhabditis,* including members of the Rhabditina (e.g. *Oscheius* spp.), Diplogastrina or Aphelenchida.

### Distribution of *Caenorhabditis* species at different spatial scales

**Figure 3:**
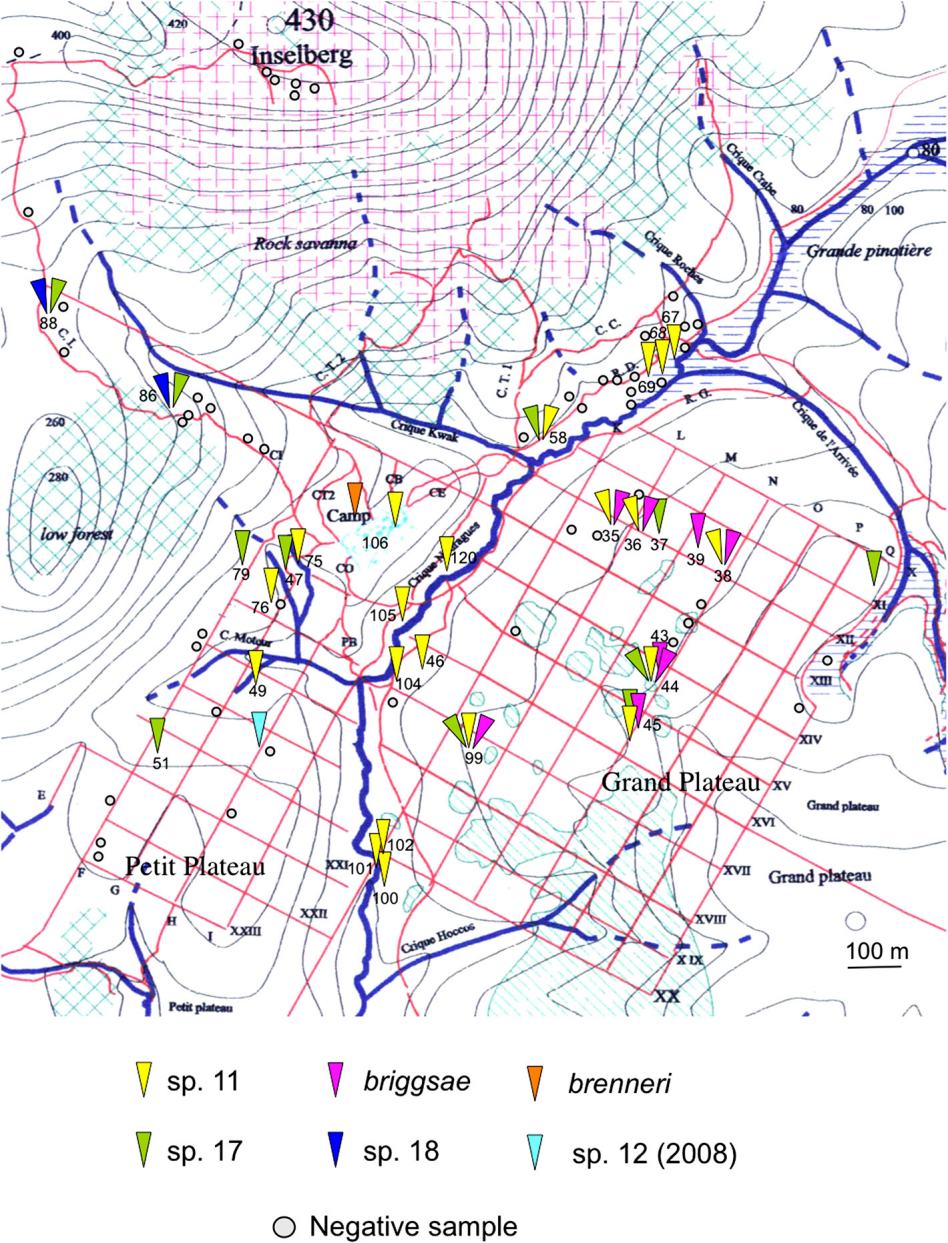
**Spatial distribution of *Caenorhabditis *species in the Nouragues Natural Reserve. **Triangles indicate the presence of a *Caenorhabditis* species, colour-coded as indicated in the legend. Numbers next to the triangles correspond to the GPS coordinate identifier (see Additional file 2). Small grey dots indicate spots containing only negative samples, i.e. samples lacking *Caenorhabditis.*

At the kilometre scale, the spatial distribution of *Caenorhabditis* species appears non-uniform across the landscape in the Nouragues Natural Reserve (Figure 3), suggesting that geographic and ecological factors structure species distributions at this medium scale. Indicative of potential species structuring are the following observations: 1) all seven of the *C. briggsae* isolations were obtained from the Grand Plateau area, 2) the 11 samples that contained any *Caenorhabditis* from the Nouragues river area (a swamp wood called “pinotière”, i.e. dominated by *Euterpe oleracea* palms) included *C*. sp. 11 (and in one instance also *C.* sp. 17), suggesting that *C*. sp. 11 is more common than other *Caenorhabditis* species in this habitat, and 3) *C.* sp. 17 was found commonly over the entire sampling area, except perhaps in the river area.

To test for small-scale (i.e. geographically localised) spatial structuring of species and genotype diversity in the Nouragues Natural Reserve, we focused on two sampling sites (H3 and H6) that contained large numbers of rotting *Cecropia* fruits on the ground (Additional file 2, Figure 4). At site H3, we processed 52 samples, of which 23 contained *C. briggsae* (7 of 11 fruit samples, 1 of 3 leaf litter samples, 2 of 4 invertebrate samples) and one contained *C*. sp. 11 (a fruit sample without *C. briggsae*). At site H6, we processed 47 samples, of which 15 contained *C. briggsae* (6 of 11 fruit samples, 1 of 3 leaf litter samples, 1 of 3 humus samples) and 4 contained *C.* sp. 11 (one fruit sample without *C. briggsae*; co-occurring with *C. briggsae* in 3 fruit samples). At both sites, many substrate samples (60 of 99) also contained other nematode species, including *Oscheius* spp., which co-occurred with the *Caenorhabditis* species in 16 of 38 cases.

**Figure 4:**
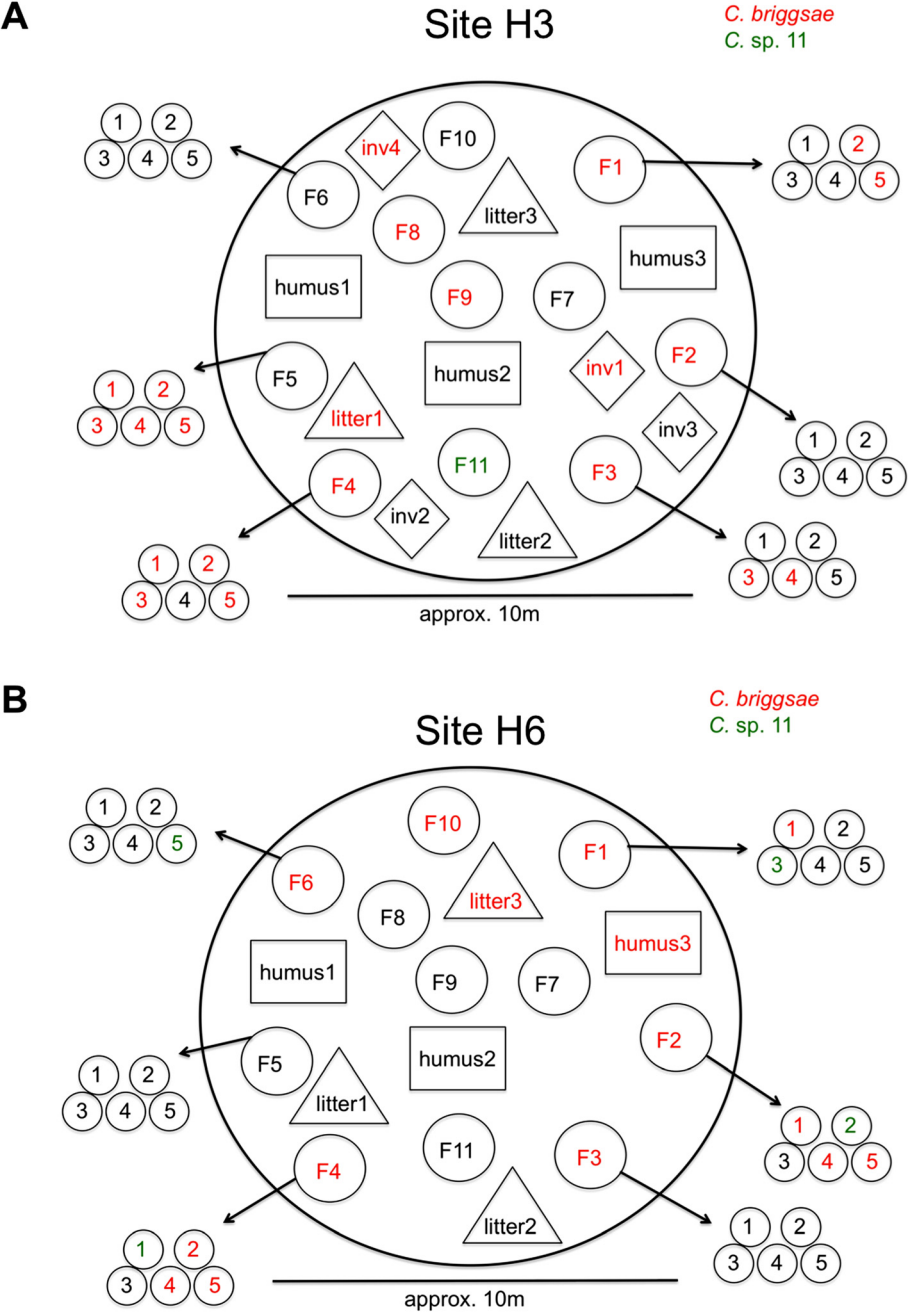
**Small-scale spatial sampling of *Cecropia *fruits in Nouragues, French Guiana. **Two independent sites, H3 (**A**) and H6 (**B**) were sampled. F = fruit, with subsampling of five individual "fingers", numbered 1 to 5 (see *Cecropia* fruit morphology in Additional file 1). In addition, leaf litter and humus were sampled at the same sites, as well as invertebrates ("inv"), such as snails.

### Pattern of *C. briggsae* genetic diversity at different spatial scales

To test for local population structure within a species, we focused on the *C. briggsae* isolates resulting from the above small-scale spatial analysis, then considered these together with isolates from the entire Nouragues area and from other sampling sites in French Guiana. We further placed the results in the context of worldwide diversity of this species.

**Figure 5:**
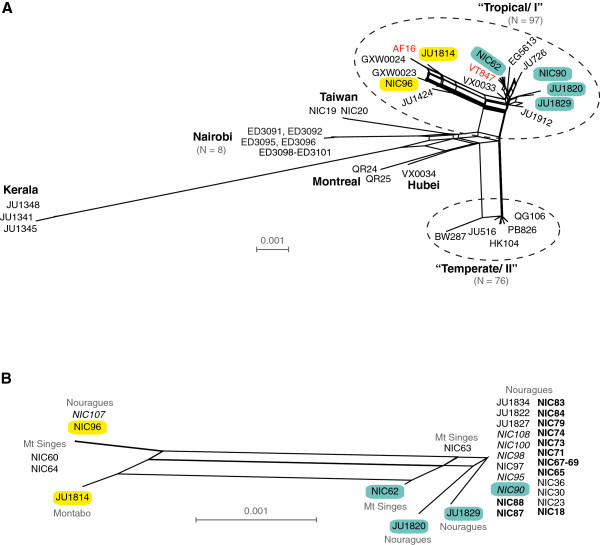
**Neighbour-network based on the concatenated sequences of six nuclear loci depicting the relationships among *C. briggsae *isolates isolated worldwide (A) and from French Guiana (B). **(**A**) Pattern of global *C. briggsae* diversity from a worldwide collection of 189 isolates (57 new from this study). Selections of isolates are labelled for reference; reference isolates AF16 and VT847 are indicated in red. Reticulation indicates potential recombination among isolates. (**B**) French Guiana isolates are partitioned into two groups that do not reflect the spatial distribution between the sampling sites but that correspond to the more general clustering observed within the “Tropical/I.” A subset of isolates is colour-coded similarly (yellow or blue) on both panels for reference. Isolates in bold derive from the subsampling of *Cecropia* H3, those in italics of H6. Note that only isolates with sequence information across the set of six loci were used to infer relationships among isolates.

We first investigated the global pattern of *C. briggsae* diversity with a multi-locus dataset of 189 isolates, including 57 new isolates derived primarily from tropical sites (Additional files 3 and 4). Our broad-scale analysis (Figure 5A) is consistent with previous reports indicating that most *C. briggsae* isolates partition into two main groups that largely separate by latitude, the “Tropical/I” and “Temperate/II” phylogeographic groups, in addition to a few genetically distinct isolates from geographically-restricted localities that do not follow this latitudinal dichotomy [6,16-20]. Genetic differentiation among the phylogeographic groups is very strong in relative terms (*F*_*ST*_ > 0.85; Table 2), although the absolute magnitude of divergence between them is modest (*D*_*xy*_ < 1.6%) owing to the low species-wide polymorphism in *C. briggsae*. This contrasts with the weaker *F*_*ST*_ and greater absolute divergence among populations in species such as *C. remanei*[21]. Here, we merge prior naming schemes for genetic groups within *C. briggsae* that were based on phylogeography with those numerical designations used for mitochondrial haplotype groups, e.g. “Tropical” as in [6] versus “clade I” as in [20], both for clarity with previous literature and to retain biologically informative labels when referring to genetic groups of *C. briggsae* for which there is evidence of local adaptation [22].

**Table 2:** **Differentiation (*F***_***ST***_**, below diagonal) and per-site divergence (*D***_***xy***_**, above diagonal) between phylogeographic groups for the 6 nuclear loci combined**

	**Tropical/I**	**Temperate/II**	**Nairobi**	**Kerala**	**Taiwan**	**Montreal**
Tropical/I (n = 97)		0.0047	0.0056	0.0159	0.0059	0.0052
Temperate/II (n = 76)	0.854		0.0068	0.0167	0.0069	0.0063
Nairobi (n = 8)	0.880	0.989		0.0150	0.0047	0.0063
Kerala* (n = 3)	0.936	0.975	0.972		0.0159	0.0155
Taiwan* (n = 2)	0.867	0.974	0.962	0.967		0.0064
Montreal (n = 2)	0.877	0.993	0.994	0.976	0.978	

*Only the highly differentiated strains are labelled “Kerala” and “Taiwan” (Figure 5A).

The genetic composition of several isolates further demonstrates that demarcation of the range boundary for the two main haplotype groups within *C. briggsae* does not strictly adhere to the Tropic of Cancer (Figure 5A) [19,20]. Specifically, Chinese isolates GXW0023 and VX0033 from Wuhan (30°N) and GXW0024 from Weifang (36°N) clearly fall into the “Tropical/I” haplotype group, but are found farther north than most such isolates, and farther north even than some isolates from the “Temperate/II” haplotype group. Despite these exceptions, 93.6% of 141 “Tropical/I” isolates were collected in tropical or subtropical latitudes, and 100% of 72 “Temperate/II” isolates were collected in temperate latitudes. Interestingly, in addition to the more common “Tropical/I” isolates, the sets from Kerala, Hubei and Taiwan each included strains corresponding to distinct haplotype groups, suggesting that sampling at local scales might reveal substantial genetic diversity [16,19]. This observed diversity motivated us to investigate local patterns of diversity of the *C. briggsae* isolates from French Guiana.

All *C. briggsae* isolated from French Guiana are nested within the "Tropical/I" phylogeographic group. The multi-locus analysis showed that they cluster into two sub-groups that mirror the pattern of differentiation observed circum-globally for “Tropical/I” isolates (“AF16-like” and “VT847-like;” Figure 5). The *C. briggsae* isolates from French Guiana thus harbour a great part of the genetic diversity found within "Tropical/I," despite the relatively small region sampled.

Within French Guiana, these two genetic sub-groups do not partition according to locality of origin. Indeed, diverse genotypes occurred at the two sites where several *C. briggsae* isolates were collected (i.e. both Nouragues and Montagne des Singes). In addition, we found little differentiation (*F*_*ST*_ = 0.267) and divergence (*D*_*xy*_ = 0.0020) between isolates from Nouragues and Montagne des Singes. Moreover, genotypes from the two genetic subgroups co-occurred at the smallest scale in the sub-sampling of *Cecropia* fruits (H6; italics in Figure 5B). Distinct genotypes also occurred at the scale of 5–10 metres in Montagne des Singes (NIC63 vs. NIC64). This result indicates that population structure among the French Guiana genotypes is not consistent with strong local population genetic differentiation that would lead to isolation by distance. Instead, the pattern of population genetic diversity of *C. briggsae* within French Guiana is consistent with it being a microcosm of pan-tropical diversity patterns, at least for the level of resolution permitted with single nucleotide polymorphism in nuclear loci. However, one multilocus genotype was dominant in the samples from Nouragues, which likely contributes to its non-zero differentiation with samples from Montagne des Singes (Figure 5B).

To further investigate local patterns of diversity at various spatial scales, we then compared nucleotide polymorphism of *C. briggsae* that were collected in French Guiana with isolates from several other locations. Although different genotypes were found on the same fruit in French Guiana (Figure 5B), we did not observe any nucleotide variation within a sample of isolates collected in New Jersey at a scale of a few metres (Table 3). This result is consistent with the finding that “Temperate/II” isolates harbour thirteen-fold less nucleotide polymorphism (per site π_si_ = 0.00012), on average, than isolates from “Tropical/I” (π_si_ = 0.00159; Table 3) [6]. At an intermediate spatial scale, we found overall similar levels of nucleotide diversity between the *C. briggsae* isolates from French Guiana and isolates from the island of Maui, Hawaii (Table 3). Nevertheless, it is noteworthy that the size of the sample from Maui is six-fold smaller than that of French Guiana. Further intensive sampling of small islands is warranted to investigate total diversity in *C. briggsae*, which might uncover novel sequence variants. For instance, inclusion of *C. briggsae* isolates from several Hawaiian islands raises global measures of nucleotide polymorphism (Table 3).

**Table 3:** **Population genetic summary statistics for different sample sets of *C. briggsae*, averaged across six nuclear loci**

Origin*	**Sample type**^**a**^	**n**^**b**^	**Hd**^**c**^	**h**^**d**^	**S**^**e**^	**π**_**si**_**(%)**^**f**^	**θ**_**si**_**(%)**^**g**^
New Jersey	Local	13.8	0	1.0	0	0	0
Nouragues	Local	42.7	0.123	2.8	3.7	0.045	0.132
All French Guiana	Pooled	50.2	0.249	3.3	4.0	0.089	0.140
Maui	Local	8.0	0.355	2.0	2.2	0.179	0.133
All Hawaii	Pooled	12.3	0.452	2.8	3.8	0.214	0.203
Tropical/I	Pooled	127.5	0.425	7.3	7.2	0.159	0.234
Temperate/II	Pooled	77.7	0.034	1.8	1.7	0.012	0.076
Worldwide	Pooled	221.5	0.564	11.3	20.3	0.403	0.691

*See Figure 5A; ^**a**^“Local” samples refer to isolates collected from a single putative population whereas “pooled” samples refer to isolates collected from disjoint populations [23,24]; ^b^n: mean number of strains; ^c^Hd: haplotype diversity; ^d^h: number of haplotypes; ^**e**^S: number of segregating sites; ^**f**^π_si_: average number of nucleotide differences per silent site; ^**g**^θ_si_: nucleotide diversity per silent site.

## Discussion

### High *Caenorhabditis* species richness in a tropical rainforest

Here we documented the extensive sampling of *Caenorhabditis* in a tropical rainforest in French Guiana and characterized the structure of genetic diversity of the cosmopolitan species *C. briggsae* from small (~1 m), intermediate (~1 km) and up to global spatial scales. We identified six species from a single tropical site, representing nearly 25% of the 26 *Caenorhabditis* species known in laboratory culture [3]. Three of these species may be endemic to this region, although sampling of *Caenorhabditis* in South America is very limited. These findings reinforce the view that *Caenorhabditis* nematodes can be locally diverse and – although they have been generally referred to as soil nematodes – are more easily found in rotting vegetal matter, especially fruits, flowers and stems [3,13].

Our sampling can be contrasted with a recent extensive sampling effort in temperate France [13]. In mainland France, just two species of *Caenorhabditis* are found commonly: *C. elegans* and *C. briggsae*. In addition, *C. remanei* and *C.* sp. 13 each have been isolated once in the course of sampling many locations in France over many years [13]. The isolation of six distinct *Caenorhabditis* species in just a few square kilometres of tropical forest, including three potentially endemic species, testifies to much higher species richness of *Caenorhabditis* in the tropics [3]. Moreover, this finding for *Caenorhabditis* is fully consistent with latitudinal diversity patterns documented for many other taxa [25,26]. As discovery of new species in this group is progressing rapidly, phylogenetically-informed analysis of *Caenorhabditis* species distributions may shed light on long-standing questions about the causes of latitudinal species diversity gradients [27].

Previous sporadic sampling of *Caenorhabditis* nematodes in Tropical South and Central America has yielded *C.* sp. 11, *C. brenneri,* and *C. briggsae*. An additional three species have also been found on Caribbean islands (*C*. sp. 14, *C*. sp. 19, and *C*. sp. 20) [3]. The absence of the well-known *C. elegans* in the Nouragues Natural Reserve is not surprising, however, given that this species is rare in previous sampling from the tropics [3]. Thus, the distribution of *Caenorhabditis* species around the world is consistent with there being a few cosmopolitan species and many other species endemic to particular geographic regions.

Our study demonstrates the first evidence of heterogeneous species distributions of *Caenorhabditis* at an intermediate spatial scale on the order of kilometres. It will be important in future work to determine whether these species distributions reflect stable features that might correspond to distinct ecological habitat characteristics of the different species, or instead reflect stochastic local abundances that are dynamic in time, perhaps owing to strong metapopulation dynamics associated with ephemeral food patches that can be utilized by many *Caenorhabditis* species. Co-occurrence of multiple species was common for samples of the same substrate type, and sometimes within the same sample, a pattern also reported for the less species-rich sampling in mainland France [13]. These observations raise the neglected issue of niche space in *Caenorhabditis* biology as a major question to be answered to understand the abundance and distribution of species. Unfortunately, there are few data describing *Caenorhabditis* fitness reaction norms in relation to potential ecological factors that could differentially limit species persistence (e.g. temperature, bacterial food specificity, substrate chemistry) [13,22,28-30].

### Spatial structure of genetic diversity

We used the cosmopolitan *C. briggsae* as a focal taxon for quantifying population genetic variability from the finest sampling scales within the Nouragues forest samples up to pooled samples at regional and global scales. We found that distinct *C. briggsae* genotypes co-exist at scales down to a few metres (Montagne des Singes) or even a single fruit (Nouragues, sample H6). Given their highly selfing mode of reproduction in nature [6,13], this suggests that a given fruit can be colonized by multiple individuals of distinct genetic backgrounds. These results mirror findings for temperate latitude samples of *C. elegans*, in that high local diversity may occur while genetic differentiation among locations is low [8,10,11,14,31,32]. This pattern of large-scale homogeneity coupled with fine-scale heterogeneity also is reminiscent of the 'chaotic genetic patchiness' observed for some intertidal invertebrates with a highly dispersing planktonic stage [33]. In contrast to *C. elegans*, however, strong population structure dominates the global scale in *C. briggsae*, with 1) a large "Tropical/I" phylogeographic group distributed widely in the tropics around the world, 2) a large "Temperate/II" phylogeographic group having very low polymorphism overall and hence undetectable local diversity, and 3) a few genetically divergent clades, so far each found only in one place (Nairobi, Kenya; Montreal, Canada; Tai-an, Taiwan; Hubei, China; Kerala, India) [16,19]. It is likely that such geographically restricted diversity is not yet exhausted by our sampling, nor are the possibilities for observing recombination among the main phylogeographic groups. Recombination had been previously noted at this large scale [6,20] and can also be inferred among the French Guiana multilocus genotypes, as visualized by the reticulation pattern of the haplotype network (Figure 5B).

## Conclusions

In this study, we described the first sampling of *Caenorhabditis* in a primary tropical forest environment, analysing a variety of substrates. We found six *Caenorhabditis* species, mostly in rotting flowers and fruits, the densest richness of species known in this group. Three of these species are cosmopolitan, three appear less widespread and might be endemic to French Guiana. The genetic structure of *C. briggsae* populations indicates events of long-distance migrations among different continents in the tropical zone, and a local spatial structure resembling that described for *C. elegans* in temperate zone populations.

## Methods

### Sampling sites in French Guiana

Samples were collected between November 17 and 27, 2009 at the end of the dry season. Most samples were collected at the CNRS “Inselberg” field station in the Nouragues Natural Reserve (4.08809°N, -52.6796°W) within an area of approximately four square kilometres (Figure 3). We also collected samples at other locations, including mangrove forests in the outskirts of Cayenne (Montabo trail), secondary forests (e.g. Montagne des Singes, Kourou), and agricultural areas around the village of Cacao (Figure 1).

### Sample collection and analysis

We collected a variety of rotting plant material, primarily targeting rotting fruits and flowers. Such substrates are known to frequently harbour *Caenorhabditis*[3] (Additional files 1 and 2). Samples were collected, photographed and stored in plastic zip bags. A given sample identifier code refers to a substrate sample (e.g. a single or multiple fruits) at a single location of a few square metres, contained within a single bag. Part of the samples were analysed at the Nouragues field station using a dissecting microscope and ready-to-use 35 mm *C. elegans* Normal Growth Medium (NGM) culture plates previously seeded with *E. coli* OP50 [34], allowing us to determine developmental stages and number of individuals present at the time of isolation. The other samples were stored for the duration of the field trip (14 days) and examined afterwards in the laboratories in Nice and Paris, using 55 and 90 mm culture plates. Samples were spread around the lawn and 1–2 ml of water or M9 solution was added to the samples. All plates were examined regularly using the dissecting microscope, and nematodes were isolated over a period of seven days. Note that all *C. briggsae* isolates used for genetic analysis (see below) were derived from independent substrate samples, except for one sample (G2) from which eight *C. briggsae* isolates were derived from different individuals, immediately after isolation on site (isolates NIC23, NIC28, NIC29, NIC30, NIC36, NIC37, NIC38, NIC40) (Additional file 2). This sampling protocol ensured that any potential genetic differences found among isolates indicate the presence of genetically distinct natural isolates in the same substrate sample.

### Species identification and isolate establishment

*Caenorhabditis* genus identity was confirmed by microscopy using morphological criteria [34]. Species identity was further examined through mating tests with known species and/or ITS2 sequencing as described previously [3]. (Note that newly discovered species are attributed a number before a formal taxonomic description has been published). Stock cultures were established by isolation of a single hermaphrodite or female. For gonochoristic species, we picked either a female with a copulatory plug, or one female and one male. The mode of reproduction was determined by isolating virgin L4 females/hermaphrodites and scoring for the absence/presence of progeny. For androdioecious species, isogenic strains (referred to as “isolate”) were produced by isolating a single larva for a few (3–6) generations. For gonochoristic species, strains were established as isofemale lines. From each isolated *Caenorhabditis* sample, we established cultures, which were cryopreserved and stocked in our collections.

A complete list of isolates can be found in Additional file 2. JU isolates are stored in the lab of MAF and NIC isolates are stored in the lab of CB. All worm strains are available upon request.

### Geographically localised (“small-scale”) sampling of *Cecropia* fruits in the Nouragues forest

To examine the species distribution and genetic diversity of *C. briggsae* at different spatial scales, we collected a large number of samples from rotting fruits of *Cecropia* sp. (likely *Cecropia sciadophylla*) at two sites in the Nouragues Natural Reserve from which we had previously collected *C. briggsae* and other *Caenorhabditis* species (Sample ID H3, H6; Additional file 2). The forest ground at these two sites was covered by numerous rotting *Cecropia* fruits of black or sometimes brown colour. At each site, we collected 11 individual rotting fruits from the ground within an area of approximately 40 m^2^ (fruits were separated from each other by approx. 2–8 m). From six of these fruits, we derived sub-samples by collecting separately five small fruit parts ("fingers") from each fruit. From both sampling sites, we also collected three litter samples (consisting primarily of rotting leaves) and three humus samples. For one of the sites (samples H3), we further collected four invertebrate samples: one live snail, two snail shells, and one live myriapod. Collection of independent subsamples of each substrate sample on site allowed subsequent analysis to detect potential mixing of different genotypes in the same sample. For details of sampling design, see Figure 4.

### Additional *C. briggsae* isolates analysed

We further included 46 new *C. briggsae* isolates, mostly collected from rotting vegetation, from localities across five continents; including eight regions not previously sampled for *Caenorhabditis* nematodes (Additional file 3). Each isolate was founded from a single hermaphrodite individual, maintained using standard protocols, and taxonomic status was determined by mating tests and/or ITS2 sequencing [3]. The geographic and ecological information of the wild isolates was also entered in Wormbase (http://www.wormbase.org). An interactive map of collection localities and isolate information can be found online (*C. briggsae*: https://www.google.com/maps/ms?msa=0&msid=117700919974655793194.00046c7ccc0afccc319b7&t=h&z=2).

### DNA amplification and sequencing

Gene fragments corresponding to the six loci used in previous studies of *C. briggsae* diversity were amplified and sequenced using published primers [6]. DNA was isolated from large populations of isogenic worms of each strain using the DNeasy Blood and Tissue kit (Qiagen). Amplifications were processed in 30 μL reaction volumes with 1.5 μL DMSO, 3 μL dNTPs (6.6 mM), 3 μL 10X Buffer (Fermentas), 2.4 μL MgCl_2_, 0.36 μL of each primer (50 μM), 0.18 μL of Taq polymerase (New England Biolabs) and 2 μL of genomic DNA. Cycling conditions were: 95°C for 4 min followed by 35 cycles of 95°C for 1 min, 55°C or 58°C for 1 min and 72°C for 1 min. Amplifications were sequenced at the University of Arizona UAGC sequencing facility. All markers were sequenced on both strands and all polymorphisms were visually verified using sequencing chromatograms. Primer sequences were manually deleted from each sequence prior to analysis. All new sequences are accessible in GenBank with accession numbers JX288123-JX288761.

### Sequence analyses

We analysed a total of 99 new wild isolates of *C. briggsae* (53 from French Guiana, 46 from other world-wide localities). Note that four French Guiana isolates (NIC88, NIC106, NIC107, NIC108) were analysed in Jovelin & Cutter [19]. Based on a set of six nuclear loci used previously [6,16,17,19], we investigated the relationships of the 57 new isolates for which data were collected for all six loci, relative to a collection of 132 isolates that were used in previous studies on *C. briggsae* diversity [6,16,17,19]. We also obtained sequence data for up to five of these loci for the remaining new isolates, which informed some analyses (Additional file 3). Multiple sequence alignments were constructed for each locus in BioEdit [35] and manually edited for indel polymorphisms. We inferred the relationships among isolates with concatenated sequences from the set of six loci using unrooted neighbour-networks generated with a Jukes-Cantor distance in the program SplitsTree 4.10 [36]. Neighbour-networks are useful for representing the relationships among individuals of the same species for which recombination events may be non-negligible [37]. We then measured differentiation and divergence between phylogeographic groups using *F*_*ST*_[38] and *D*_*xy*_, the average number of nucleotide substitutions per site between groups [39]. We also measured nucleotide polymorphism [39] at each locus taken separately using DnaSP v.5 [40] and took the average across loci in order to investigate nucleotide diversity at multiple spatial scales in *C. briggsae*.

## Competing interests

The authors declare that they have no competing interests.

## Authors’ contributions

MAF and CB collected the French Guiana samples, and analysed them with the help of CF. ECA collected and analysed the Maui samples. RJ, SH and YRC collected sequence data and RJ analysed them. CB, MAF, RJ and ADC wrote the manuscript. CB, MAF, ADC designed and supervised the research. All authors read and approved the final manuscript.

## Supplementary Material

Additional file 1 **Photographs of substrate samples from French Guiana containing *Caenorhabditis. ***Description: From left to right and top to bottom: *Heliconia* flower, rotting *Cecropia* fruits, *Ficus* sp. (sample A1), rotten *Eperua falcata* flowers (sample D34), *Clusia grandiflora* fruits, rotten flowers (sample D14), *Inga* sp. fruit (F9), yellow fungus (sample D10). Most of the photographed fruits and flowers were less decayed than those sampled (with the exception of *Heliconia*, where the rotting matter inside erect flowers was collected). Click here for file

Additional file 2 **List of samples from French Guiana along with corresponding isolate and species analysis (three sheets). **Description: The first sheet “Nouragues” lists all samples collected at the Nouragues field station. These samples were mostly analysed on site within a few hours after isolation, allowing for *Caenorhabditis* population census and developmental stage assessment. The column “GPS ID” correspond to GPS reference numbers reported on the map in Figure 3. The column “Date sampling” indicates the day when the substrate sample was collected and the column “Sample analysis” indicates the time point at which samples were placed on culture plates for analysis. The column “*Caenorhabditis* population 12 hrs after” describes population census and developmental stages observed (only for samples that were examined on site, immediately after isolation); L1-L4: first to fourth larval stage, ad: adult. Species and isolate derived from a given sample are listed in the last column “Species and Strains”. The second sheet “*Cecropia* subsampling” lists all samples collected for the detailed geographically localised (“small-scale”) sampling of *Caenorhabditis* nematodes in rotting *Cecropia* fruits at two localities (H3 and H6) at the Nouragues Field station. For details on sampling scheme, see Figure 4 and the Materials and Methods section. The third sheet “French Guiana” details all samples collected outside the Nouragues area in other parts of French Guiana. None of these samples were analysed on site. In all sheets, positive samples, i.e. samples containing *Caenorhabditis*, are highlighted with an orange background. Click here for file

Additional file 3 **List of new *C. briggsae *isolates used to investigate nucleotide diversity. **Description: The first sheet “*C. briggsae* isolates” lists all new *C. briggsae* isolates used to examine nucleotide diversity (N=99; 53 from French Guiana, 46 from other world-wide localities). Note that four French Guiana isolates (NIC88, NIC106, NIC107, NIC108) were already analysed in Jovelin & Cutter [19] and are therefore not listed. For detailed sampling information on the previously examined *C. briggsae* isolates (N=132), see [6,16,17,19]. The last column indicates the list of sequenced loci for each isolate. Only isolates with sequence information across the set of six loci were used to infer relationships among isolates, while all isolates were used to investigate patterns of nucleotide diversity. The coloured background indicates all isolates from a same subsampling in Nouragues: H3 subsampling (yellow), H6 subsampling (green), and isolates derived from a single substrate sample, G2 (blue). The second sheet “Primers” indicates primer information and corresponding genomic regions from [6,38]. Click here for file

Additional file 4 **Haplotype structure of *C. briggsae *isolates from a worldwide distribution based on six nuclear loci. **Description: Only the polymorphic sites and indels are included. All indels are shown by a dash, regardless of length. Dots represent nucleotides identical to those shown in the top sequence. Gene names are indicated in the bottom panel with linkage groups (LG) indicated in the top panel. The number of isolates along with the isolate name is shown on the right for each haplotype. Newly sequenced isolates are labelled in bold. Click here for file
