# Supplementary figures and images for: Species richness, distribution and genetic diversity of Caenorhabditis nematodes in a remote tropical rainforest
Source: BMC Evol Biol. 2013 Jan 12;13:10. doi: 10.1186/1471-2148-13-10 (PMC3556333; doi:10.1186/1471-2148-13-10)

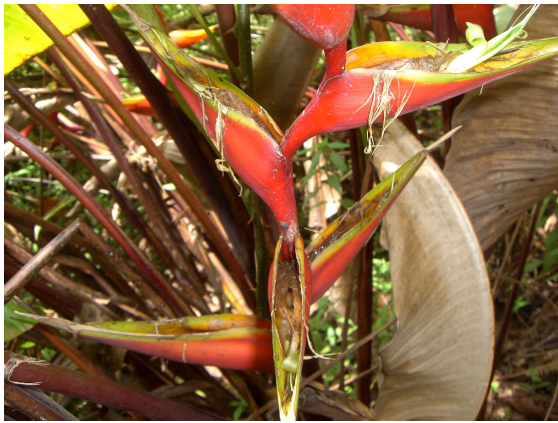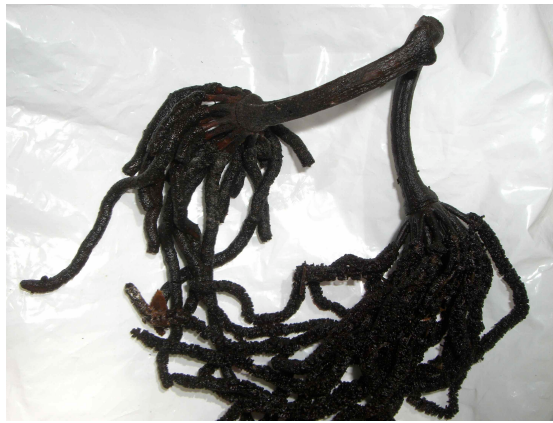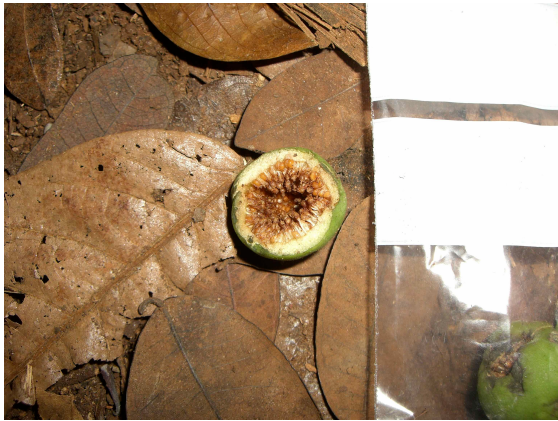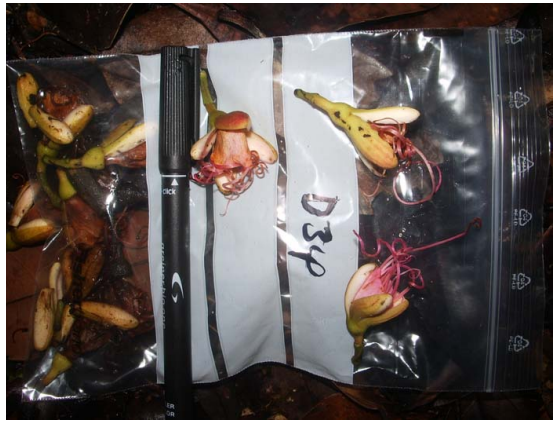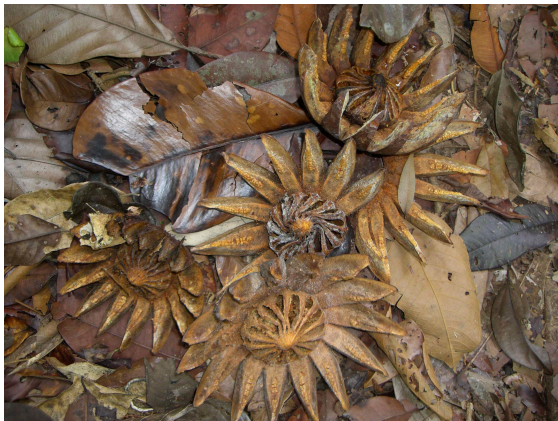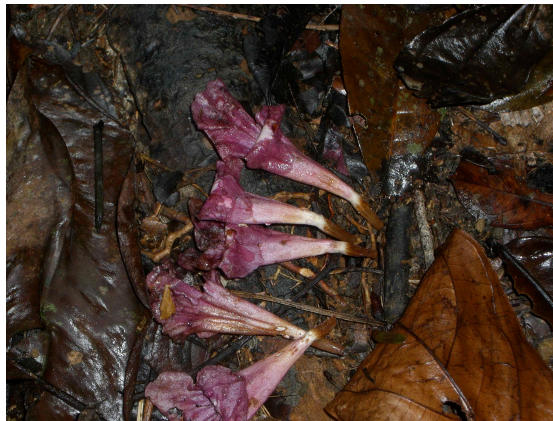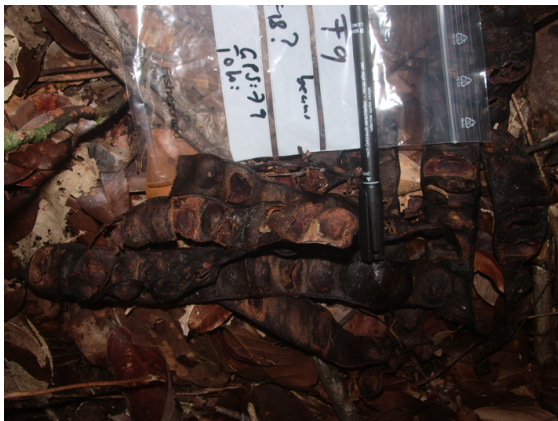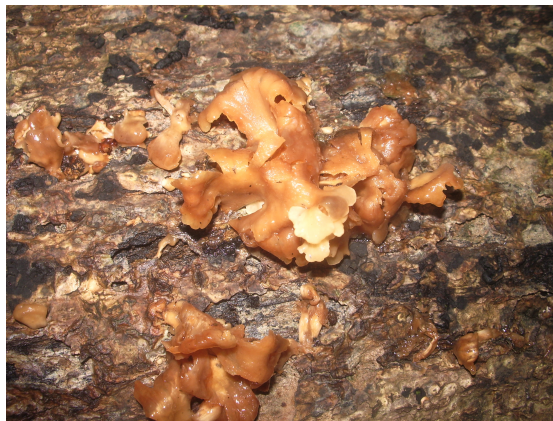

Supplement: Additional file 1 — Photographs of substrate samples from French Guiana containing Caenorhabditis. Description: From left to right and top to bottom: Heliconia flower, rotting Cecropia fruits, Ficus sp. (sample A1), rotten Eperua falcata flowers (sample D34), Clusia grandiflora fruits, rotten flowers (sample D14), Inga sp. fruit (F9), yellow fungus (sample D10). Most of the photographed fruits and flowers were less decayed than those sampled (with the exception of Heliconia, where the rotting matter inside erect flowers was collected). [file 1471-2148-13-10-S1.pdf]

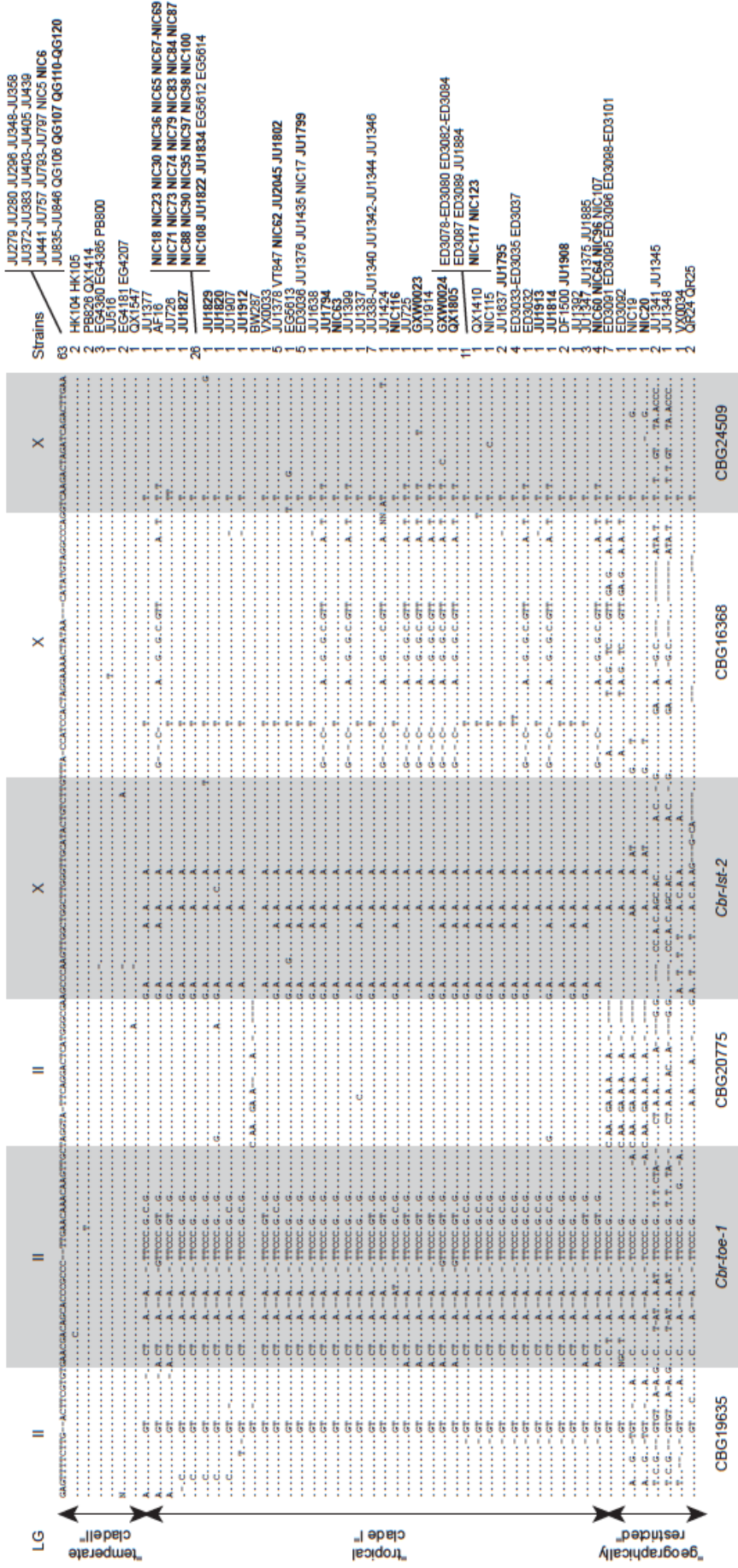

Supplement: Additional file 4 — Haplotype structure of C. briggsae isolates from a worldwide distribution based on six nuclear loci. Description: Only the polymorphic sites and indels are included. All indels are shown by a dash, regardless of length. Dots represent nucleotides identical to those shown in the top sequence. Gene names are indicated in the bottom panel with linkage groups (LG) indicated in the top panel. The number of isolates along with the isolate name is shown on the right for each haplotype. Newly sequenced isolates are labelled in bold. [file 1471-2148-13-10-S4.pdf]
